# Annotations

**Published:** 1904-12-24

**Authors:** 


### Annotations.

### Self-Supporting Colonies for the Feeble-Minded.

The Royal Commission on the Care of the Feeble-Minded sat on Monday for the last time before the Christmas holidays. The principal witness was Mr. J. G. Legge, his Majesty's Inspector of Reformatory and Industrial Schools. He declared himself strongly in favour of large colonies for the feeble-minded, and expressed himself satisfied that they would be, to a great extent, self-supporting, and thus the rates would be saved from very serious burdens. This, he thinks, would be absolutely impossible if colonies of less than 800 persons were formed. In any legislation which it may be decided to propose hereafter, the question of cost will certainly have to be considered, for the ratepayers are already called upon to pay about as much as they can without finding the strain intolerable. Mr. Legge is strongly in favour of establishing colonies where agricultural work is at hand. This is really a matter of vital importance. Mr. Legge recommends it both on the ground of physical benefit to the members of the colony, and also because they might be useful to the country side. The first of these reasons is quite sufficient. Miss Dendy, whose experiences as one of the honorary officials of the Lancashire and Cheshire Society for the Permanent Care of the Feeble-Minded give weight to her testimony, made one proposal in her voluminous evidence which is in advance of public opinion. She urged the statutory prohibition of marriage on the part of persons of feeble mind. This might be for the advantage of future generations, but there are practical difficulties in the way of carrying it into effect which will doubtless prevent the Commission from endorsing her recommendation. The evidence has so far been very interesting, and has thrown a good deal of light on the operation of the Act of 1899. Whether it will suffice to give rise to a demand for the conversion of a permissive into a compulsory measure remains to be seen.

### The Paris Sanitary Convention.

The Local Government Board has just issued a translation, by Dr. Theodore Thomson, one of the medical inspectors of the board, of the complete text of the International Sanitary Convention of Paris, 1903. Dr. Thomson was himself one of the British delegates at the International Conference at which the Convention was made, and he has thus approached his work as a translator under the great advantage of possessing detailed knowledge of the views and intentions of his colleagues. Notwithstanding this, he has found it necessary to leave certain words as they stood in the French original, on account of the difficulty or impossibility of defining the exact meaning to be attached to them. One of these is "foyer," concerning which we read in the Convention itself that, "when the cases of cholera constitute a "foyer," the local area shall be declared infected." Dr. Thomson explains in a note that the expression "centre of dissemination" may be taken as a fair equivalent for "foyer," but that it seems desirable, in view of the difficulty of deciding what should be regarded as a "foyer" of cholera, to retain the original term. The question was debated at some length at the Dresden Conference of 1893, and again at Paris by the translator himself and others. At Dresden, Professor Brouardel, one of the French delegates, said that an exact definition of the word "foyer" was a difficult matter, and it seems to have been retained in default of a better one. In one or two other matters it has been found necessary to give detailed explanations; but, as a rule, the Convention is perfectly clear, and it contains a full account of the general provisions with regard to plague or cholera which are to be observed by the countries signing it, of the measures of defence to be taken by other countries against territories that have been declared infected, of the special provisions to be enforced regarding countries outside Europe, and of the provisions regarding pilgrimages. The fourth part of the Convention embraces questions of administration and control; and the fifth part is a recommendation to the countries concerned to modify their sanitary regulations in such fashion as to bring them into harmony with the present scientific data as to the manner in which yellow fever is transmitted, and, in particular, as to the part played by mosquitoes in carrying the germs of the disease.

### The Royal Colleges of Physicians and Surgeons and their Members.

The style and title attached to membership either of the Royal College of Physicians of London or the Royal College of Surgeons of England carry, no doubt, suggestions of considerable dignity and influence. However well founded such a conclusion may be in regard to the lay public it is entirely the reverse of the truth in the collegiate world itself. Strange as it may seem to the uninitiated, it is a simple statement of fact to say that in the policy and direction of these institutions their respective members have neither part nor lot. It is true that once a year, by the grace of the Council, the members of the Royal College of Surgeons are permitted to meet and to discuss various matters in which they are interested, but they have no share in the control or direction of the College, and no representation on the governing council. The resolutions which they pass at the annual meeting are transmitted to the Council, but are entirely destitute of binding authority. Hence it is little wonder to find that, after many years of assembling to pass resolutions which produce no effect, members seem to be losing confidence in the value of the annual meeting. Such a conclusion can hardly be avoided in face of the fact that on the last occasion, out of a constituency numbering more than 17,000, the necessary quorum of 30 was counted with difficulty. Either members have lost all interest in their "rights," or they have concluded that the annual meeting offers no chance of the attainment of their demands. The College of Physicians keeps its members in an even more remote position, for it does not grant them so much as the barren privilege of an annual meeting. Evidently the strong wind of democracy has not yet reached the governing authorities of the great medical corporations.

